# Editorial: Statistical Learning for Predicting Air Quality

**DOI:** 10.3389/fdata.2022.898643

**Published:** 2022-05-05

**Authors:** Yves Philippe Rybarczyk, Rasa Zalakeviciute

**Affiliations:** ^1^School of Information and Engineering, Dalarna University, Falun, Sweden; ^2^Grupo de Biodiversidad Medio Ambiente y Salud, Universidad de Las Américas, Quito, Ecuador

**Keywords:** machine learning, urban pollution, deep learning, chemical transport model (CTM), forecast

The concentration of air pollutants is traditionally explained by complex physical and chemical processes of dispersion and advection. This is the reason why the prediction of air quality is usually addressed through deterministic models, such as Chemical Transport Models (CTMs).

However, the CTMs show several limitations and constraints. Their performance depends on an updated emission inventory of the urban area, which is often compromised in developing countries. They also struggle to make an accurate air pollution forecast in complex terrain regions. Moreover, they require high computational power, in order to run time-consuming simulations.

More recently, statistical models based on Machine Learning (ML) algorithms have appeared as a valuable alternative to tackle many disadvantages of the CTMs. They seem particularly relevant to provide a fine resolution at an urban scale, where the estimation of air contamination is of the most importance for health concerns. In that sense, ML could become the new paradigm for pollution forecasting.

The main goal of this Research Topic is to understand if ML can become the new standard for air quality prediction. Among the several ML methods, we intend to identify the most suitable algorithms for atmospheric pollution forecasting. Such an investigation considers all the dimensions of the prediction performance, which includes both the accuracy and the interpretability of the models. For example, the non-linear models (e.g., ensemble learning or artificial neural networks) tend to be more accurate but less interpretable than a linear regression.

The first paper highlights the fact that a data-driven method such as ML can consider an infinite number of factors affecting air quality, which can improve drastically the prediction. Saheer et al. explain that ML can take into account several heterogenous factors, such as urban traffic, aerial imagery of terrains and vegetation, and weather conditions, for a more reliable prediction of air quality. The authors propose a cost-effective framework composed of different machine learning methods, from statistical to deep learning algorithms.

The benefit of ML over the CTM approach is demonstrated in the second article. Fan et al. compare the performance of a chemical transport model (AIRPACT) and two machine leaning (ML) models to forecast O_3_ in Kennewick (WA, USA). The first ML model (ML1) uses the random forest (RF) classifier and multiple linear regression (MLR) models, and the second model (ML2) uses a two-phase RF regression model. ML1 and ML2 are the best models to predict high and low O_3_ pollution events, respectively. On top of that, the ML models require much less computational resources than AIRPACT, which suggests that ML is a better solution than CTMs to forecast O_3_.

The third study shows that ML is not only a suitable method to predict O_3_ but can be applied to predict any kind of pollutants. Mendes et al. use Classification and Regression Tree (CART) and multiple regression (MR) to forecast PM_10_, PM_2.5_, NO_2_, and O_3_ concentrations in Portugal (Lisbon and Madeira) and Macao. The proposed models are able to predict the concentration of the pollutants for the next day, with a good accuracy.

Finally, the last manuscript addresses the question of the effect of the COVID-19 Lockdown on air quality change. Chau et al. propose a new approach based on Weather Normalized Modeling to get a more reliable estimation of the concentration of pollutants under a business-as-usual assumption. Several Deep Learning (DL) algorithms and Gradient Boosted Machine (GBM) are tested to quantify the impact of the human mobility reduction on the concentration of the criteria pollutants (CO, NO_2_, PM_2.5_, SO_2_, and O_3_) in Quito, Ecuador. The results show that Long-Short Term Memory (LSTM) and Bidirectional Recurrent Neural Network (BiRNN) outperform the other algorithms. All the pollutants have significantly reduced, except O_3_ that increased by titration effect. Besides revealing the better accuracy of DL over the other methods, this work identifies the most important factors to predict air pollution.

Overall, the studies of this Research Topic tend to demonstrate that statistical or machine learning is a powerful alternative method to the traditional CTM approach, whatever the aspect of pollution forecasting considered. ML is a fast and affordable technique which requires less computational power for an accuracy that can be higher than CTM. Also, the recent progress in the ML algorithms allow a disclosure of the models, which were until now considered as a black box. Resolving the model interpretation issue can definitely rank the ML approach as the best method for predicting air quality.

## Author Contributions

YR has written the article. RZ has revised and edited the text. All authors contributed to the article and approved the submitted version.

## Conflict of Interest

The authors declare that the research was conducted in the absence of any commercial or financial relationships that could be construed as a potential conflict of interest.

## Publisher's Note

All claims expressed in this article are solely those of the authors and do not necessarily represent those of their affiliated organizations, or those of the publisher, the editors and the reviewers. Any product that may be evaluated in this article, or claim that may be made by its manufacturer, is not guaranteed or endorsed by the publisher.

